# Educator Knowledge of Childhood Conduct Problems and Callous-Unemotional Traits

**DOI:** 10.1007/s10802-024-01230-9

**Published:** 2024-07-13

**Authors:** Georgette E. Fleming, Antonia L. Boulton, Ashneeta H. Prasad, Kelly A. Kershaw, Eva R. Kimonis

**Affiliations:** Parent-Child Research Clinic, School of Psychology, The University of New South Wales, Sydney, NSW Australia

**Keywords:** Conduct problems, Callous-unemotional traits, Limited prosocial emotions, Mental health literacy, Educators, Teachers

## Abstract

Research evaluating mental health literacy (MHL) of adults who support children with mental health difficulties is relatively scarce. To date, no studies have investigated educator knowledge of conduct problems and callous-unemotional (CU) traits. This is a significant gap in the literature since conduct problems are among the most prevalent childhood mental disorders, while CU traits are associated with poor academic, behavioral, and social outcomes in school settings. In the current study, we assessed educators’ knowledge of the characteristics and management of conduct problems and CU traits. Participants were *N* = 390 preschool and primary/elementary school educators (*M*_age_ = 38.62 years, *SD* = 11.66; 91% woman-identifying; 71% White) who completed a Knowledge Test and survey assessing educator characteristics and various student-educator outcomes. Averaged across items, educators scored 57.1% on the Knowledge Test. We identified gaps in educator knowledge with respect to identifying characteristics associated with distinct domains of externalizing difficulties and evidence-based management strategies. Educators’ years of experience and accreditation status were not associated with knowledge. Paraeducators had significantly lower knowledge scores than teachers and leadership. Unexpectedly, greater knowledge was not associated with better student-teacher relationship quality or more positive perceptions of students with conduct problems. Findings support the need for universal MHL programs focused on conduct problems and CU traits, especially among paraeducators, while also suggesting that more intensive interventions may be required to improve educator-student relationship quality.

**Supplementary Information:**

The online version contains supplementary material available at 10.1007/s10802-024-01230-9.

A significant proportion of mental health conditions onset during the early childhood period (Kim-Cohen et al., 2003). Early intervention is essential to prevent negative outcomes associated with stable and persistent behavioral and socioemotional difficulties. Critically, children are often unable or unwilling to seek help for mental health conditions. Instead, adults usually facilitate referral and treatment processes on behalf of the child (Stiffman et al., 2004). While caregivers are well positioned to seek help for children, preschool and elementary school educators also play a key role in the help-seeking processes (Johnson et al., 2023). During early schooling, children spend a significant portion of the week with a stable teaching team, who are thus ideally placed to recognize children who experience persistent behavioral or socioemotional difficulties. This ability largely relies on accurate knowledge about mental health conditions. Knowledge is the keystone of mental health literacy (MHL), defined by Jorm and colleagues (1997, p. 182) as “the knowledge and beliefs about mental disorders that aid in their recognition, management or prevention.” As a concept, MHL has received considerable attention in relation to adult and adolescent mental disorders, but research focusing on MHL of adults who support young children with mental disorders is scarcer (Johnson et al., 2023; Tully et al., 2019). Moreover, most of this limited work has focused on adults’ knowledge and recognition of Attention-Deficit/Hyperactivity Disorder (ADHD), while some has considered internalizing disorders, Autism Spectrum Disorder (ASD), or mental disorders generally (Johnson et al., 2023). Notably, few studies have exclusively focused on conduct problems or their key diagnoses, Oppositional Defiant Disorder (ODD) and Conduct Disorder (CD). We aim to address this gap by examining knowledge of conduct problems among preschool and elementary school educators.

Conduct problems represent a constellation of acting-out behaviors that fall on a continuum from emotional outbursts and defiance to behaviors that violate social norms or others’ rights, such as aggression and property destruction (American Psychiatric Association [APA], 2013; Kimonis & Fleming, 2018). The scarcity of research investigating adults’ knowledge of childhood conduct problems is significant given ODD and CD are among the most prevalent childhood mental disorders, with a combined worldwide prevalence of 5.7% in youth aged 6–18 years (Polanczyk et al., 2015) and 4.5% in children aged ≤ 7 years (Vasileva et al., 2021). Childhood conduct problems have detrimental impacts, especially when they onset during the early childhood period and persist over time (Bevilacqua et al., 2018). Of particular relevance, early-onset conduct problems are associated with significant risk of poor educational outcomes, including academic underachievement, placement in high-needs education classes, and school dropout (Bevilacqua et al., 2018; Bierman et al., 2013). They also predict poor social outcomes at school, including social skills deficits, relational aggression, peer rejection, deviant peer affiliation, student-teacher conflict, and low likelihood of positive classroom climates (Bardach et al., 2022; Brennan et al., 2015). As such, school-based conduct problems not only impact individual children, but also affect the safety, wellbeing, and educational experiences of their peers and teachers. Accordingly, it is vital that educators are equipped to identify persistent disruptive behaviors and initiate a referral to evidence-based support (Splett et al., 2019). Importantly, conduct problems often occur within classroom settings, so educators also play a key role in managing disruptive behaviors. Thus, educators’ MHL must extend beyond identification and referral to include knowledge about evidence-based principles and strategies of behavior management.

To date, there has been limited empirical investigation of whether educators have sufficient knowledge to identify and manage children with conduct problems. The few studies examining MHL in relation to conduct problems have focused on ODD, assessing educators’ concern about, recognition of, and degree of self-efficacy managing ODD-related behaviors. These studies are consistent in finding that educators tend to express more concern about hypothetical students showing disruptive behaviors, ODD, or externalizing problems than those with internalizing problems, likely because of the impact these students have on classroom climate and safety (Loades & Mastroyannopoulou, 2010; Splett et al., 2019). However, other research suggests that educators may struggle to recognize ODD, with one study of pre-service Kindergarten-Grade 12 teachers in Canada (*N* = 186) finding that fewer than half (47.9%) correctly identified ODD in a vignette about an 8-year-old student, compared to 84.4% and 89.6% of teachers who correctly identified ADHD and anxiety, respectively (Whitley & Gooderham, 2016). More markedly, a study of middle and high school teachers in the United States (*N* = 462) found that only 0.8% of teachers correctly identified ODD in a vignette of an age-unspecified student, relative to 56.7% of teachers who correctly identified depression (Green et al., 2022). Finally, relative to vignettes of children with ADHD, anxiety, and depression, teachers reported the lowest self-efficacy managing a student with ODD (Whitley & Gooderham, 2016). Taken together, these studies indicate that teacher knowledge of ODD appears to be lower than for other common childhood mental disorders (i.e., ADHD, internalizing disorders). Moreover, while teachers are concerned about students with ODD, they report lower self-efficacy managing ODD compared to these other disorders. Critically, no study quantitatively assessed teachers’ knowledge about classroom management strategies for ODD.

A final key limitation of this literature is that it ignores evidence for distinct subtypes of children with conduct problems. In particular, no study has evaluated educators’ knowledge of conduct problems that co-occur with callous-unemotional (CU) traits. Subtyping according to the presence of CU traits has proven useful for designating a subgroup of children with early-onset, stable, severe, and pervasive conduct problems (Frick et al., 2014; Neo & Kimonis 2021). This subtyping approach was codified in the *Diagnostic and Statistical Manual of Mental Disorders* (5th ed.; DSM-5; APA, 2013) as a specifier for Conduct Disorder (CD) called ‘with limited prosocial emotions’ (LPE), given when a child meets criteria for CD and also shows two or more of the following symptoms persistently across multiple settings or relationships: [a] lack of remorse or guilt, [b] callous-lack of empathy, [c] lack of concern about performance, and [d] shallow/deficient affect. According to the *International Classification of Diseases and Related Health Problems* (11th ed.; ICD-11; World Health Organization, 2019), this specifier can also be given when a child meets criteria for ODD. The lack of attention to children with conduct problems and CU traits in the MHL literature is significant because this subtype is associated with considerable risk for poor outcomes even among children with conduct problems, including the highest rates of delinquency, substance-use, and psychopathy (Frick et al., 2014). With respect to educational outcomes, compared to their peers with conduct problems alone, children with CU traits have poorer academic engagement (e.g., Hwang, Waller et al., 2021) and achievement (e.g., Horan et al., 2016) and worse behavioral outcomes, including higher rates of aggression and bullying (e.g., Ciucci et al., 2014; Crum et al., 2016). CU traits are also associated with social deficits at school, including lower teacher- and self-rated social competence (Haas et al., 2018), lower peer-rated social preference, and poorer student-teacher relationships (e.g., Crum et al., 2016; see Levine et al., 2022 for review).

The importance of educators’ ability to recognize conduct problems that co-occur with CU traits is underscored by evidence that CU traits impact the effectiveness of educators’ standard classroom behavior management strategies. In particular, teachers rate students with CU traits as less sensitive to punishment than their non-CU peers (e.g., Allen et al., 2018; Baroncelli et al., 2022), with some evidence that punishment insensitivity accounts for the relationship between CU traits and poor academic achievement (Hwang, Allen et al., 2021). Research also suggests that CU traits (but not conduct problems) predicted reduced teacher-delivered rewards over time (Hwang et al., 2020), with some inconsistent evidence that CU traits are associated with lower sensitivity to specific types of rewards (e.g., social; Allen et al., 2018). Importantly, CU traits also appear to impact the student-teacher relationship, such that elevated CU traits are associated with more conflict and less closeness in the relationship above and beyond conduct and anxiety problems (Bégin et al., 2021; Horan et al., 2016), but especially when CU traits co-occur with conduct problems (Crum et al., 2016). Thus, students with CU-type conduct problems present a particular challenge to educators in classroom settings. Teacher knowledge about CU traits thus appears important, especially insofar as educators are required to adapt management strategies to accommodate the unique temperamental, cognitive, and socioaffective needs of students with CU traits.

## The Current Study

Accordingly, the current study aimed to address these gaps in the literature by evaluating educators’ knowledge about conduct problems and CU traits. Given the importance of early identification and intervention to prevent antisocial trajectories (Bevilacqua et al., 2017), we invited educators who work with preschool- and elementary school-aged children (ages 3 to 12 years) to participate. Our first aim was to understand educators’ knowledge of the characteristics of conduct problems and CU traits, along with evidence-based principles and strategies of behavior management for conduct problems. Our second aim was to understand how educator characteristics are related to knowledge. We hypothesized that greater knowledge would be associated with more years in the profession, higher accreditation status, and status as a classroom teacher or leadership compared to school learning support officer or aide (i.e., ‘paraeducators’). Our third aim was to understand the associations between educator knowledge and educator self-efficacy, student-teacher relationship quality, and educator perceptions of student conduct problems. We hypothesized that greater knowledge would predict greater self-efficacy; more closeness and less conflict in the student-teacher relationship; and less endorsement of student conduct problems as intentional, dispositional, and permanent.

## Method

### Participants

Participants were *N* = 408 preschool and elementary school educators who commenced a survey prior to participating in a universal MHL program focused on student conduct problems. Of these, *n* = 18 participants were excluded due to incompletion (*n* = 17) or ineligibility due to being a high school teacher (*n* = 1), leaving a final sample of *N* = 390 educators. Table 1 presents the demographic characteristics of the sample. Educators were aged between 20 and 70 years and most self-identified as women (91%) and White (71%). The sample was thus broadly representative of Australian educators, most of whom were women (76%), born in Australia (83%, racial/ethnic identity not reported), and aged 30–59 years (70%) in 2020 (AITSL, 2023). Educators were eligible to participate in the study if they were a teacher or paraeducator for preschool or elementary-school aged children educated in Australia. Educators were ineligible to participate if they planned on completing any other mental health training related to conduct problems in the upcoming three months.

**Table 1:** Demographic characteristics of educators

Variable	M (SD)
Educator Age (years)	*N* = 387 38.62 (11.66) Range: 20.86-70.00
	* N (%)*
Gender	*N* = 390
Woman	355 (91.0%)
Man	33 (8.5%)
Prefer not to answer	2 (0.5%)
Race / Ethnicity	*N* = 390
White	277 (71.0%)
Asian	39 (10.0%)
Middle Eastern	35 (9.0%)
Aboriginal or Torres Strait Islander	9 (2.3%)
African	2 (0.5%)
Pacific Islander	1 (0.3%)
Other	27 (6.9%)
Years Experience	*N* = 390
0–5 years	147 (37.7%)
6–10 years	74 (19.0%)
11–15 years	55 (14.1%)
16–20 years	36 (9.2%)
21–25 years	24 (6.2%)
25–30 years	25 (6.4%)
30 + years	29 (7.4%)
Accreditation Status	*N* = 382
Unaccredited	32 (8.4%)
Provisionally/conditionally accredited	93 (24.3%)
Proficient/highly accomplished/lead	257 (67.3%)
Current Role	*N* = 231
Teacher	157 (68.0%)
Leadership	38 (16.5%)
Paraeducator	36 (15.6%)
Geographic Location	*N* = 390
Metropolitan	363 (93.1%)
Regional	25 (6.4%)
Rural	2 (0.5%)
Type of Institution	*N* = 390
Government/public	376 (96.4%)
Systemic (e.g., Catholic)	2 (0.5%)
Independent/non-government	5 (1.3%)
Other	7 (1.8%)
Institution Setting	*N* = 390
Early childhood	13 (3.3%)
Elementary	377 (96.7%)

### Procedure

This study was approved by the UNSW Human Research Ethics Committee (HC17078/HC220077), Human Research Advisory Panel (3275), and NSW State Education Research Applications Process (SERAP2017152). The participants were recruited as part of an ongoing larger study examining the effects of universal and targeted intervention for parents and educators of children with conduct problems (ACTRN12619000967189). Educators completed the MHL training either online (*n* = 142) or face-to-face with a clinically-trained facilitator (*n* = 248). Educators who participated online were recruited via email invitations sent to government, systemic, and independent elementary schools in New South Wales, Australia, to complete the training as part of a school-wide formal professional learning plan or a self-directed professional learning activity. Educators who participated face-to-face were recruited via a network of seven pre/elementary institutions involved in a trial of targeted school-based intervention, who completed the training as part of a pre-Grade 2 formal professional learning plan (see Table S1 [Supplementary materials] for group comparisons). Prior to participating in the training, educators were provided with information about the study and invited to consent to participate in research. Educators then completed a 30–45 min pre-training survey comprising demographic questions, a knowledge test, and a questionnaire assessing their self-efficacy. In the survey, educators were also asked whether they currently/previously worked with a student with conduct problems. Educators were free to select any student they wished. Educators who nominated a student by providing their initials (*n* = 324, 83%) then completed questionnaires assessing student-teacher relationship quality, as well as their perceptions about the intentionality, dispositionality, and permanence of the student’s conduct problems. Per our ethical approval, we did not collect any identifying demographic, developmental, or diagnostic information^1^ about the nominated student. Surveys were completed using paper or online via Qualtrics or REDCAP survey software. Educators were permitted to skip questions if they preferred not to respond. Data collection for the universal training commenced in 2021 and is ongoing. In this study, only the pre-training survey data were used.

### Measures

#### Demographic Questionnaire

We invited participants to provide their age, gender identity, race/ethnicity, years of educator experience, and accreditation status, as well as their workplace’s geographic location, type of institution, and institution setting. We also asked educators who completed the face-to-face training (*n* = 248) to specify their current workplace role (teacher, leadership, or paraeducator), although *n* = 17 did not respond to this question. Regarding accreditation status, teachers receive Conditional accreditation when they have successfully completed an undergraduate degree and are enrolled in a postgraduate teaching degree or have completed at least three years of a four-year undergraduate teaching degree. This level of accreditation allows teachers to work as a teacher while they complete their university training. Teachers receive Provisional accreditation once they have been awarded their teaching degree. Teachers receive Proficient accreditation status once they have provided evidence of meeting all the benchmarks specified in the Australian Professional Standards for Teachers (NSW Education Standards Authority, 2022).

#### Knowledge of Conduct Problems

We assessed educators’ knowledge of conduct problems using a 35-item Knowledge Test. We developed the Knowledge Test to assess educators’ understanding of the information provided in the universal MHL training, which covered three topics: (1) the characteristics of conduct problems (e.g., “Which of the following symptoms are all associated with Conduct Problems?”), including CU traits (e.g., “Which of the following symptoms are associated with Limited Prosocial Emotions”); (2) evidence-based theory and principles of behavior management (e.g., “Positive Reinforcement refers to:…”); and (3) evidence-based behavior management strategies (e.g., “What types of rewards are most effective for motivating appropriate student behaviours?”). The training was directly informed by principles and strategies from Parent-Child Interaction Therapy and Teacher-Child Interaction Training (Niec, 2018). Educators responded to items on 3- to 7-point or true/false response scales, with correct scores summed to compute a total knowledge score ranging from 0 to 35, with higher scores indicative of greater knowledge. Since educators were permitted to skip questions, total scores were only computed if educators answered 33/35 questions (0.26%). In the current study, internal consistency of total knowledge scores was in the questionable range (Cronbach’s α = 0.59 / McDonald’s ω = 0.60.). We also summed the questions corresponding to each topic: characteristics of conduct problems (3 items); CU traits / LPE (3 items); principles of behavior management (10 items); and strategies of behavior management (19 items). The number of questions within each topic reflected the amount of training time and content allocated to each topic. However, it should be noted that these topic scores had very poor internal consistency (αs = 0.18–0.46 / ωs = 0.33–0.45), so results relating to these topic scores are purely descriptive. See Table S2 for all items and answers.

#### Educator Self-Efficacy

We assessed educator self-efficacy using the 12-item Teacher’s Sense of Self-Efficacy Scale– Short Form (TSSE-SF; Tschannen-Moran & Hoy, 2001). Educators rated their efficacy in student engagement (e.g., “How much can you do to calm a student who is disruptive or noisy?”), instructional strategies (e.g., “To what extent can you craft good questions for your students?”), and classroom management (e.g., “How much can you do to control disruptive behaviour in the classroom?”) on a 9-point Likert scale from 1 (*Nothing*) to 9 (*A great deal*), with scores summed to compute a total scale score. TSSE-SF total scores range from 12 to 108, with higher scores indicative of higher self-efficacy. In the current study, internal consistency of total TSSE-SF scores was excellent (α = 0.94 / ω = 0.94).

#### Student-Teacher Relationship Quality

We assessed student-teacher relationship quality using the 15-item Student-Teacher Relationship Scale– Short Form (STRS-SF; Pianta, 2001). Educators rated the level of closeness (e.g., “I share an affectionate, warm relationship with this student “) and conflict (e.g., “This student and I always seem to be struggling with each other”) in their relationship with their nominated student on a 5-point Likert scale from 1 (*Definitely does not apply*) to 5 (*Definitely applies*). Scores were summed to compute 8-item Closeness and 7-item Conflict scales. Closeness and Conflict scales range from 8–40 and 7–35, respectively, with lower scores on the Closeness scale and higher scores on the Conflict scale indicative of lower student-teacher relationship quality. In the current study, internal consistencies of STRS-SF Closeness and Conflict scales were acceptable (α =.83 and.75 / ω =.83 and.76).

#### Educator Attributions about Student Conduct Problems

We assessed educators’ perceptions about students with conduct problems using an adapted version of the 12-item Parent Attribution Measure (PAM; Sawrikar et al., 2019), called the Teacher Attribution Measure (TAM). Educators rated their belief in the intentionality (e.g., “The student ‘pushes my buttons’ on purpose”), dispositionality (e.g., “I worry that the student is a bad person”), and permanence (e.g., “The student will always be a problem”) of their nominated student’s conduct problems on a 3-point Likert scale from 1 (*Not at all true*) to 3 (*Certainly true*), with scores summed to compute 4-item Intentionality, Disposition, and Permanence scales. The three scales range from 4 to 12, with higher scores indicative of a tendency to perceive students’ conduct problems more negatively (i.e., as intentional, dispositional, and permanent). In the current study, internal consistencies of the TAM Intentionality, Disposition, and Permanence scales were questionable to acceptable (α = 0.73, 0.62, and 0.65 / ω = 0.74, 0.62, and 0.67).

### Planned Analyses

#### Educator Knowledge of Conduct Problems

To address aim 1, we calculated the percentage each answer was endorsed for each item on the Knowledge Test. We also calculated the average accuracy percentage across items for the total Knowledge Test and each knowledge topic, as well as provided a description of the items most and least likely to be answered correctly within each topic.

#### Educator Characteristics and Educator Knowledge of Conduct Problems

To address aim 2, we calculated the total number of correct items on the Knowledge Test for each educator. We then conducted a series of one-way ANOVAs using the UNIANOVA procedure in SPSSv29 to examine whether total number of correct items on the Knowledge Test differed according to years of experience as an educator (0–5 vs. 6–10 vs. 11–15 vs. 16–20 vs. 21–25 vs. 26–30 vs. 30 + years), accreditation status (unaccredited vs. provisionally/conditionally accredited vs. accredited), and current role (paraeducator vs. teacher vs. leadership). For each ANOVA, we tested two planned contrasts. For teaching experience, we were interested in whether educators with more experience interacting with students with conduct problems had greater knowledge than educators with less experience, so we tested early (0–5 years) vs. mid (6–15 years) career and early (0–5 years) vs. established (16–30 + years) career educators. For accreditation status, we were interested in whether educators who had undertaken the professional development necessary to be provisionally or fully accredited had greater knowledge than unaccredited educators, so we tested unaccredited vs. provisionally/conditionally accredited and unaccredited vs. fully accredited educators. Finally, for current role, we were interested in whether educators who had a university qualification and practical training had greater knowledge than less qualified or trained educators, so we tested paraeducator vs. teacher and paraeducator vs. leadership. We applied a Bonferroni correction, such that differences were considered significant at α = 0.025.

#### Educator Knowledge of Conduct Problems and Student-Teacher Outcomes

To address aim 3, we conducted a series of linear regressions using the REGRESSION procedure in SPSSv29 to examine whether total number of correct items on the Knowledge Test was associated with educator self-efficacy; closeness and conflict in the student-teacher relationship; and educators’ attributions regarding the intentionality, dispositionality, and permanence of student conduct problems. We checked assumptions for all ANOVA and regression models.

## Results

### Educator Knowledge of Conduct Problems

Averaged across all items, educators achieved 57.1% (*SD* = 27.27) accuracy on the Knowledge Test. We report the endorsement percentage of each answer option for all 35 Knowledge Test item in Table S2. When we calculated average accuracy for each topic of the Knowledge Test, we found that educators achieved 62.2% (*SD* = 29.02) accuracy on items assessing their knowledge of *characteristics of conduct problems*. The item least likely to be answered correctly assessed educators’ knowledge of the symptoms of conduct problems. Educators were more likely to endorse an answer that included “hyperactivity/impulsivity” (46.8%) rather than “deceitfulness/lying” (30.6%) in a list of symptoms associated with conduct problems, while a proportion (16.7%) also identified “lack of remorse/guilt,” “lack of empathy,” and “poor social skills” as symptoms associated with conduct problems. Educators achieved 59.2% (*SD* = 33.63) accuracy on items assessing their knowledge of *CU traits*, with just over half of educators (50.1%) accurately identifying the CU/LPE criteria. Fewer educators accurately identified the behavioral goal that was least relevant for students with CU traits, with only 31.0% identifying “improved self-confidence” as the correct response. Educators achieved 67.6% (*SD* = 26.50) accuracy on items assessing their knowledge of *evidence-based principles of behavior management*. The items least likely to be answered correctly assessed educators’ understanding of negative reinforcement (20.0%), positive punishment (29.0%), and negative punishment (45.4%), although most educators correctly understood positive reinforcement (93.1%). Finally, educators achieved 50.4% (*SD* = 26.85) accuracy on items assessing their knowledge of *evidence-based strategies of behavior management*. The items least likely to be answered correctly assessed educators’ understanding of child-led play strategies, including the role of questions in play (4.9%) and the definition of negative talk in a play context (28.7%); the characteristics of effective instructions (6.2 to 27.7%); and the ratio of positive to negative attention when establishing a positive student-teacher relationship (29.3%).

### Educator Characteristics and Educator Knowledge of Conduct Problems

Bivariate correlations between main study variables are presented in Table 2. Regarding years of experience, we found that total knowledge scores (*M* = 20.62, *SD* = 3.82) did not significantly differ between educators with 0–5 (*M* = 20.52, *SD* = 4.00), 6–10 (*M* = 20.93, *SD* = 3.70), 11–15 (*M* = 20.54, *SD* = 3.83), 16–20 (*M* = 21.31, *SD* = 4.01), 21–25 (*M* = 20.13, *SD* = 3.37), 26–30 (*M* = 19.60, *SD* = 3.70), or 30+ (*M* = 21.00, *SD* = 3.43) years of experience, *F*(6, 382) = 0.71, *p* =.64, η_p_^2^ = 0.01. Neither of the planned contrasts comparing experience groups were significant (Table 3). This pattern of results did not change when we reran the analysis with *n* = 6 outliers removed.

**Table 2:** Pearson zero-order correlations between main study variables

	1	2	3	4	5	6	7	8	9	10	11	12
1. Knowledge	-											
2. Years of experience^a^	− 0.01	-										
3. Accreditation status^b^	0.05	0.42**	-									
4. Current role^c^	− 0.15*	− 0.06	− 0.58**	-								
5. TSSE self-efficacy	0.14*	0.18**	0.30**	− 0.26**	-							
6. STRS closeness	0.04	− 0.004	0.04	− 0.07	0.17**	-						
7. STRS conflict	0.06	− 0.01	0.09	− 0.09	− 0.03	− 0.30**	-					
8. TAM intentionality	− 0.04	− 0.09	− 0.01	− 0.02	− 0.11*	− 0.35**	0.48**	-				
9. TAM disposition	− 0.002	0.04	− 0.002	0.08	− 0.10*	− 0.40**	0.48**	0.55**	-			
10. TAM permanence	0.13*	0.04	0.08	0.08	− 0.04	− 0.16**	0.26**	0.23**	0.40**	-		
11. Educator age	− 0.07	0.76**	0.20**	0.22**	− 0.01	− 0.02	− 0.05	− 0.10*	0.01	− 0.002	-	
12. Educator gender^d^	0.01	0.14**	0.04	0.02	0.01	0.08	0.004	− 0.05	− 0.07	0.03	0.15**	-

*Note* TSSE = Teacher’s Sense of Self-Efficacy Scale– Short Form; STRS = Student-Teacher Relationship Scale– Short Form; TAM = Teacher Attribution Measure

*p <.05, **p <.01

^a^Years of experience coded: 1 = 0–5 years, 2 = 6–10 years, 3 = 11–20 years, 4 = 16–20 years, 5 = 21–25 years, 6 = 26–30 years, 7 = 30 + years

^b^Accreditation status coded: 0 = Unaccredited, 1 = Provisionally/conditionally accredited, 2 = Accredited

^c^Current role coded: 0 = Teacher, 1 = Leadership, 2 = Paraeducator

^d^Gender coded: 1 = Man, 2 = Woman, 3 = Non-binary (nil data), 4 = I use a different term (nil data), 5 = Prefer not to answer

Regarding accreditation status, we found that total knowledge scores did not significantly differ between unaccredited (*M* = 19.25, *SD* = 4.15), provisionally/conditionally accredited (*M* = 21.12, *SD* = 3.80), or accredited (*M* = 20.70, *SD* = 3.71) educators, *F*(2, 379) = 2.91, *p* =.056, η_p_^2^ = 0.02. The contrast comparing unaccredited and provisionally/conditionally accredited educators was significant (Table 3), such that unaccredited educators scored significantly lower on the Knowledge Test than provisionally/conditionally accredited educators. The contrast comparing unaccredited and accredited educators was not significant using α = 0.025. Regarding assumptions, although Shapiro-Wilk tests indicated a normality violation for the accredited group, visual inspection of the Q-Q plot indicated that this non-normal distribution was not significant. With outliers removed (*n* = 5), the omnibus test became significant (*p* =.024), while the contrast comparing unaccredited and accredited educators remained non-significant (*p* =.03). We elected to interpret the original analysis since the outlying scores represented meaningful educator responses.

**Table 3:** Contrast estimates, significance levels, and effect sizes for ANOVA planned contrasts

	Contrast	Contrast estimate	Lower 95% CI	Upper 95% CI	*p*	η_*p*_^2^
*Years Experience*						
0–5 years (early) vs.	6–15 years (mid)	0.22	-0.70	1.13	0.64	0.001
	16–30 + years (established)	-0.01	-0.95	0.94	0.98	< 0.001
*Accreditation Status*						
Unaccredited vs.	Prov/cond accredited	1.86	0.34	3.38	.0166*	0.02
	Accredited	1.45	0.06	2.84	0.041	0.01
*Current Workplace Role*						
Paraeducator vs.	Teacher	2.22	0.76	3.68	0.003*	0.04
	Leadership	2.56	0.72	4.40	0.007*	0.03

*Note* CI = Confidence Interval; prov/cond = provisionally/conditionally; Paraeducator = school learning support officer or aide

*p <.025

Regarding current workplace role, we found that total knowledge scores significantly differed between paraeducators (*M* = 18.36, *SD* = 4.37), teachers (*M* = 20.58, *SD* = 3.98), and leadership (*M* = 20.92, 3.77), *F*(2, 227) = 5.05, *p* =.007, η_p_^2^ = 0.04. Both planned contrasts were significant (Table 3), such that paraeducators scored significantly lower than teachers and leadership. This pattern of results did not change when we reran the analysis with *n* = 2 outliers removed.

### Educator Knowledge of Conduct Problems and Student-Teacher Outcomes

The model predicting educator self-efficacy (*M* = 86.31, *SD* = 11.98) was significant, *F*(1, 386) = 7.77, *p* =.006, R^2^ = 0.02. Consistent with our hypothesis, higher knowledge scores were significantly associated with higher self-efficacy scores, *B* = 0.05, *SE(B)* = 0.02, β = 0.14, *p* =.006 (95% CI 0.01, 0.08). This pattern of results did not change when we reran the analysis with *n* = 2 outliers removed.

The model predicting teacher-reported student-teacher closeness (*M* = 30.03, *SD* = 5.82) and conflict (*M* = 23.39, *SD* = 5.12) was not significant, *F*(2, 320) = 1.40, *p* =.25, R^2^ = 0.01. Contrary to hypotheses, higher knowledge scores were not significantly associated with greater student-teacher closeness, *B* = 0.05, *SE(B)* = 0.04, β = 0.07, *p* =.22 (95% CI -0.03, 0.12), or lower student-teacher conflict, *B* = 0.06, *SE(B)* = 0.04, β = 0.08, *p* =.16 (95% CI -0.02, 0.15), with the teacher-nominated student with conduct problems. This pattern of results did not change when we reran the analysis with *n* = 1 outlier removed.

Finally, the model predicting teacher-reported attributions about the intentionality (*M* = 6.69, *SD* = 1.85), dispositionality (*M* = 5.88, *SD* = 1.52), and permanence (*M* = 8.08, *SD* = 1.65) of student conduct problems was not significant, *F*(3, 319) = 1.85, *p* =.14, R^2^ = 0.02. Contrary to hypotheses, higher knowledge scores were not significantly associated with lower intentionality scores, *B* = -0.08, *SE(B)* = 0.14, β = -0.04, *p* =.56 (95% CI -0.35, 0.19), or disposition scores, *B* = -0.06, *SE(B)* = 0.18, β = -0.02, *p* =.76 (95% CI -0.42, 0.30). Unexpectedly, higher knowledge scores were significantly associated with higher permanence scores, *B* = 0.32, *SE(B)* = 0.14, β = 0.14, *p* =.025 (95% CI 0.04, 0.60). This pattern of results did not change when we reran the analysis with *n* = 3 outliers removed.

## Discussion

We aimed to extend the literature on MHL of adults who support children with mental health difficulties by assessing educators’ knowledge of conduct problems and CU traits. We identified some gaps in educators’ knowledge about conduct problems, especially with respect to parsing symptoms associated with conduct problems, CU traits, and ADHD, as well as evidence-based strategies for relationship-building and managing conduct problems. Contrary to hypotheses, neither years of experience nor teaching accreditation status were associated with greater knowledge overall. Consistent with hypotheses, educators employed as a teacher or leadership had significantly greater knowledge than paraeducators. Finally, we found that greater knowledge was associated with higher educator self-efficacy, but not the quality of the student-teacher relationship or educators’ perceptions of the intentionality, dispositionality, or permanence of their students’ conduct problems. We discuss each of these key findings in turn.

### Educator Knowledge of the Externalizing Dimension of Psychopathology

The results of the Knowledge Test indicated that many educators had difficulty distinguishing between symptoms of conduct problems (i.e., ODD and CD) and symptoms of ADHD (i.e., hyperactivity and impulsivity). Some educators also confused symptoms of conduct problems and CU traits (e.g., lack of guilt and empathy). On one hand, these domains of psychopathology often co-occur, falling within the broader dimension of ‘externalizing’ difficulties (Flom & Saudino, 2018). For the sake of identification of students in need of support and referral, it is likely unnecessary for educators to be able to distinguish between these diagnostic categories. On the other hand, conduct problems, ADHD, and CU traits represent distinct domains associated with specific genetic and nonshared environmental effects (Flom & Saudino, 2018); unique temperamental and attachment risk factors (Willoughby et al., 2011, 2014, 2022); and distinct socioaffective, cognitive, and behavioral correlates and outcomes (Waller et al., 2015). Overall, research provides support for multiple developmental pathways to conduct problems, whereby problems can develop via a ‘hot’ pathway characterized by emotional regulation deficits (ODD) and/or poor attentional, inhibitory, and behavioral control (ADHD), or a ‘cold’ pathway characterized by fearlessness and impairments in guilt and empathy (CU) (Frick & Morris, 2004).

Educators’ understanding of these etiological differences is important because educators are required to manage the behavior of these students on a daily basis. However, factors that maintain these behavior problems can differ between children with conduct problems, ADHD, and CU traits. Accordingly, educators’ behavior management strategies should reflect this heterogeneity in externalizing problems (Willoughby et al., 2022). For example, peer conflict is a behavioral correlate of all three domains of externalizing difficulties, but the mechanisms underpinning this difficulty can differ between domains. For children with conduct problems and/or ADHD, emotional and behavioral dysregulation may impact a child’s ability to *attend to and encode* social cues, potentially leading to peer conflict and rejection (Dodge & Pettit, 2003; Frick & Morris, 2004; Morris et al., 2021). In contrast, the empathy deficits unique to CU traits may undermine peer relationships by impacting children’s *motivation* to respond appropriately to social cues (Haas et al., 2018; Willoughby et al., 2022). Accordingly, educators may need to individualize how they manage peer conflict for each child. This personalized approach is consistent with efforts in the clinical literature to adapt parenting interventions for children with conduct problems and CU traits by targeting CU-specific risk factors, including deficits in empathy, punishment learning, and parental warmth (see Fleming, 2023). These efforts have shown promise for improving treatment outcomes among children with CU-type conduct problems, relative to standard behavioral interventions (e.g., Fleming et al., 2022).

### Educator Knowledge of Evidence-Based Management Strategies

However, findings from the Knowledge Test also indicated that educators’ knowledge of evidence-based behavior management strategies may also benefit from intervention, with an average of just over half of items answered correctly in this topic. This is consistent with educator self-report: Fewer than half endorsed having the knowledge or skills necessary to manage mental health needs in children (Reinke et al., 2011). Educators identified the main areas in which they needed additional knowledge or training as being strategies for working with students with externalizing difficulties and classroom behavioral management (Reinke et al., 2011). Results are also consistent with observational findings that educators do not consistently implement evidence-based practices in the classroom (State et al., 2019). Accordingly, it is critical that educators access MHL training that improves their knowledge of both the characteristics of conduct problems and evidence-based strategies for managing these problems. Promisingly, prior investigations report significant improvement in educators’ knowledge of ADHD management following MHL training, although evidence is mixed for improved student outcomes (Ward et al., 2022). Based on our findings, we suggest that MHL training for conduct problems should delineate between the characteristics, etiology, and behavioral correlates of distinct domains of externalizing difficulties, and include approaches for personalizing management strategies according to these domains.

### Importance of MHL Training for Paraeducators

Ensuring access to MHL training for conduct problems appears to be particularly important for educators employed as paraeducators. In the current study, paraeducators had significantly lower Knowledge Test scores than teachers and leadership. Since more experience was not associated with greater knowledge of conduct problems, years on-the-job may be less important for knowledge acquisition than amount of study or specialized training on this topic. The finding that conditionally or provisionally accredited educators were significantly more knowledgeable about conduct problems than unaccredited educators also supports this conclusion. In the Australian context, employment as a paraeducator does not require a teaching degree, so paraeducators were the least likely to be accredited in the current study. Moreover, evidence suggests that paraeducators do not otherwise receive appropriate training or supervision once employed (e.g., Frantz et al., 2022). Critically, however, paraeducators are the most likely to work directly with students with externalizing difficulties to implement behavior management plans (Fisher & Pleasants, 2012; Reddy et al., 2021; State et al., 2019). There is some evidence supporting the potential of MHL training to improve paraeducators’ knowledge and skills in behavior management, although controlled studies are scarce (Reddy et al., 2021). Findings from the current study underscore the importance of building this evidence base by developing and disseminating MHL training to paraeducators that incorporates strategies for personalizing behavior management.

### Targeting the Student-Teacher Relationship in MHL Training

The final key finding of the current study is that greater educator knowledge was associated with higher educator self-efficacy, but not higher educator-rated student-relationship quality or more positive attributions about student conduct problems. These findings suggest that MHL training aiming to increase educators’ knowledge about conduct problems and their management may enhance educators’ confidence, but be insufficient to shift the quality of educators’ interactions with students with conduct problems. This is significant because positive student-teacher relationships predict student academic, social, and behavioral achievement (McGrath & van Bergen, 2015; Poling et al., 2022). Accordingly, universal MHL training alone may not be enough. Educators may require a more intensive intervention that specifically incorporates strategies for improving the student-teacher relationship, alongside more ‘classic’ behavior management strategies. Promisingly, evidence supports the effectiveness of classroom management interventions that prioritize educators’ relationship-building skills for improving student-teacher relationship quality (e.g., Incredible Years– Teacher Classroom Management; Webster-Stratton et al., 2011). Overall, this is consistent with a stepped approach to supporting educators’ implementation of behavior management, and addresses existing evidence that didactic instruction alone may be insufficient to produce lasting changes in educator and student behavior (State et al., 2019).

The implementation of more intensive interventions with educators of students with conduct problems may be especially important for children with CU traits. Students with CU traits have more conflictual and less close relationships with their educators than other students (Levine et al., 2022), while low student-teacher affiliation predicts increases in CU traits over time (Hwang et al., 2022). Evidence from the parenting literature also indicates that CU traits are associated with parents’ negative attributions about the causes of their child’s difficulties, over and above conduct problems (Palm et al., 2019). Thus, any universal or targeted intervention program focused on conduct problems may benefit from including psychoeducation on the importance of relationship-building with students with CU traits, as well as practical strategies for strengthening the student-teacher relationship (e.g., child-led play) and shifting educators’ unhelpful cognitions about their student with CU traits.

### Study Limitations

Our findings should be considered within the context of important limitations. First, we only assessed two components of MHL—knowledge and recognition of conduct problems and their management—and did not assess educators’ help-seeking knowledge, attitudes, or behavior (e.g., referrals, stigmatizing beliefs), which are also important aspects of MHL (Johnson et al., 2023). Second, we assessed conduct problems as a single construct rather than assessing educators’ knowledge about ODD and CD separately. Future research examining ODD, CD, CU, and ADHD separately would likely reveal a more comprehensive and nuanced picture of educators’ knowledge, management strategies, and help-seeking in relation to externalizing difficulties.

Third, we developed our own measure of educator knowledge of conduct problems that specifically assessed knowledge of the characteristics and management of conduct problems in pre- and elementary school-aged students, as presented in a MHL training informed by PCIT and TCIT for educators. As such, it may have limited validity and generalizability beyond this study (e.g., use with secondary educators). Moreover, the total knowledge score had questionable internal consistency, although this is unsurprising given knowledge is unlikely to be a unidimensional construct, the breadth of content assessed, and differing question difficulty. On the other hand, the individual topic scores also had poor internal consistency, which precluded their use as outcomes in the current analyses, limiting our ability to explore whether specific subdomains of educator knowledge were associated with predictors and correlates of interest. To our knowledge, a comprehensive and psychometrically-robust measure of educators’ knowledge of conduct problems has yet to be developed, which is an important avenue for future research. Similarly, our measure of teacher attributions has yet to be validated, although significant small-moderate correlations between TAM and STRS-SF scales provide preliminary support for the TAM’s construct validity. Moreover, while the brevity of the TAM may be an advantage to its research and clinical utility, the small number of items comprising each subscale likely impacted internal consistency. However, the field currently lacks a comprehensive, psychometrically-sound measure of attributions for elementary teachers, which is another important research direction. Indeed, most prior research used hypothetical vignettes to assess teacher attributions for misbehavior, which fail to capture attributions for specific students (Nemer et al., 2019).

Fourth, this study relied entirely on teacher-reported questionnaire measures, subjecting it to mono-method and mono-informant bias. Future research would benefit from synthesizing information from teachers, parents, and students, as well as employing both survey and observational measures (e.g., of student-teacher relationship quality) to avoid shared method variance. Finally, consistent with our ethical approval, we did not collect identifying information about the teacher-nominated students with conduct problems, including demographic information such as sex/gender and racial, ethnic, or cultural identity, as well as information regarding diagnostic status. Accordingly, we were unable to independently verify that nominated students had clinically significant conduct problems or assess the presence of relevant comorbidities^1^, which is an important task for future research. Moreover, prior studies demonstrate that student characteristics such as race or ethnicity can moderate educators’ concern about and referrals for ODD (Green et al., 2022), so extensions of this study should also consider the predictive utility of student characteristics for teacher mental health literacy across domains of externalizing problems.

## Conclusion

Limitations notwithstanding, this study is the first to examine educator knowledge of the characteristics and management of conduct problems and CU traits. It contributes to the growing literature on educator MHL, which is important given educators’ role in identifying, referring, and intervening with students with mental health difficulties. Externalizing problems are the most common type of mental health difficulty that educators face, but teachers report having inadequate knowledge and skills to manage them effectively (Reinke et al., 2011). The results of the current study corroborate these findings, supporting the need for universal educator training on conduct problems that not only synthesizes information on the characteristics, etiology, and behavioral correlates of distinct domains of externalizing difficulties, but also incorporates methods for personalizing management strategies according to these domains. However, results also suggest that improving knowledge may be insufficient to improve educators’ relationships with their students with conduct problems, indicating that a stepped approach to educator training may be necessary when conduct problems are severe, perhaps especially when they co-occur with CU traits. Accordingly, we echo recent calls for more research funding dedicated to understanding, preventing, and treating externalizing difficulties in childhood (de Brito et al., 2021), extending this call by emphasizing the need to include educators in these critical research endeavors.

## Electronic Supplementary Material

Below is the link to the electronic supplementary material.

Supplementary Material 1

## Footnotes

1 Educators who completed the training online and nominated a student with conduct problems (*n* = 110) also reported on the nominated student’s conduct, hyperactivity, and emotional problems using the Strengths and Difficulties Questionnaire (SDQ; Goodman, 2001; see Supplementary Materials). Of these nominated students, educators rated 96% of students (*n* = 105) in the clinical range for conduct problems, 89% (*n* = 98) for hyperactivity problems, and 38% (*n* = 42) for emotional problems. Although representative of only a subset of the sample of educators (28%), these findings provide some preliminary support for the validity of the nomination method, demonstrating that educators were fairly uniformly able to identify students with externalizing problems, with evidence of possible internalizing comorbidities for some students.
