# Supplementary material for: Educator Knowledge of Childhood Conduct Problems and Callous-Unemotional Traits
Source: Res Child Adolesc Psychopathol. 2024 Jul 13;52(11):1693–706. doi: 10.1007/s10802-024-01230-9 (PMC11564242; doi:10.1007/s10802-024-01230-9)
Supplement: Supplementary file 1 — Supplementary Material 1 [file 10802_2024_1230_MOESM1_ESM.docx]

**Educator Knowledge of Childhood Conduct Problems and Callous-Unemotional Traits**

**Supplementary Materials**

**Method**

**Measures**

***Student Psychopathology***

We assessed student psychopathology for a subset of educators (*n* = 110) using subscales from the 25-item Strengths and Difficulties Questionnaire (SDQ; Goodman, 2001). Teachers rated the severity of their nominated student’s conduct (e.g., “Often fights with other children or bullies them”), emotional (e.g., “Many worries or often seems worried”), and hyperactivity (e.g., “Constantly fidgeting or squirming”) problems on a 3-point Likert scale from 0 (*Not true*) to 2 (*Certainly true*), with scores summed to compute 5-item Conduct Problems, Emotional Problems, and Hyperactivity subscales. The three subscales range from 0–10, with higher scores indicative of more severe problems. In the current study, Cronbach’s αs for each subscale was .60, .73, and .59, while McDonald’s ω was .63, .77, and .62, respectively.

**Supplementary Table 1**

*Demographic Characteristics of Educators for Overall Sample and by Training Format*

| Variable | Overall Sample | Online | In-person |  |
| --- | --- | --- | --- | --- |
|  | *M (SD)* | *M (SD)* | *M (SD)* | Significance Test |
| Educator Age (years) | *N* = 387  38.62 (11.66) | *n* = 142  38.18 (12.65) | *n* = 245  38.88 (11.05) | *U* = 18343, *z* = 0.89, *p* = .37 |
|  | *N (%)* | *N (%)* | *N (%)* |  |
| Gender | *N* = 390 | *N* = 142 | *N* = 248 | χ2(1) = 9.18, *p* = .002^a^* |
| Woman | 355 (91.0%) | 121 (85.2%) | 234 (94.4%) |  |
| Man | 33 (8.5%) | 20 (14.1%) | 13 (5.2%) |  |
| Prefer not to answer | 2 (0.5%) | 1 (0.7%) | 1 (0.4%) |  |
| Race / Ethnicity | *N* = 390 | *N* = 142 | *N* = 248 | χ2(1) = 3.57, *p* = .059^b^ |
| White | 277 (71.0%) | 109 (76.8%) | 168 (67.7%) |  |
| Asian | 39 (10.0%) | 14 (9.9%) | 25 (10.1%) |  |
| Middle Eastern | 35 (9.0%) | 6 (4.2%) | 29 (11.7%) |  |
| Aboriginal or Torres Strait Islander | 9 (2.3%) | 1 (0.7%) | 8 (3.2%) |  |
| African | 2 (0.5%) | 1 (0.7%) | 1 (0.4%) |  |
| Pacific Islander | 1 (0.3%) | 1 (0.7%) | 0 (0.0%) |  |
| Other | 27 (6.9%) | 10 (7.0%) | 17 (6.9%) |  |
| Years Experience | *N* = 390 | *N* = 142 | *N* = 248 | χ2(2) = 2.52, *p* = .28 |
| 0-5 years (early) | 147 (37.7%) | 55 (38.7%) | 92 (37.1) |  |
| 6-15 years (mid) | 129 (33.1%) | 52 (36.6) | 77 (31.0%) |  |
| 16-30+ years (established) | 114 (29.2%) | 35 (24.6%) | 79 (31.9%) |  |
| Accreditation Status | *N* = 382 | *N* = 142 | *N* = 240 | χ2(2) = 14.69, *p* = .001* |
| Unaccredited | 32 (8.4%) | 3 (2.1%) | 29 (12.1%) |  |
| Provisionally/conditionally accredited | 93 (24.3%) | 44 (31.0%) | 49 (20.4%) |  |
| Proficient/highly accomplished/lead | 257 (67.3) | 95 (66.9%) | 162 (67.5%) |  |
| Geographic Location | *N* = 390 | *N* = 142 | *N* = 248 | χ2(1) = 1.73, *p* = .19^c^ |
| Metropolitan | 363 (93.1%) | 129 (90.8%) | 234 (94.4%) |  |
| Regional | 25 (6.4%) | 12 (8.5%) | 13 (5.2%) |  |
| Rural | 2 (0.5%) | 1 (0.7%) | 1 (0.4%) |  |
| Type of Institution | *N* = 390 | *N* = 142 | *N* = 248 | χ2(1) = 0.26, *p* = .61^d^ |
| Government/public | 376 (96.4%) | 136 (95.8%) | 240 (96.8%) |  |
| Systemic (e.g., Catholic) | 2 (0.5%) | 1 (0.7%) | 1 (0.4%) |  |
| Independent/non-government | 5 (1.3%) | 5 (3.5%) | 0 (0.0%) |  |
| Other | 7 (1.8%) | 0 (0.0%) | 7 (2.8%) |  |
| Institution Setting | *N* = 390 | *N* = 142 | *N* = 248 | Fisher’s exact test = .037* |
| Early childhood | 13 (3.3%) | 1 (0.7%) | 12 (4.8%) |  |
| Primary/elementary | 377 (96.7%) | 141 (99.3%) | 236 (95.2%) |  |

*Note.* **p* < .05

^a^Gender binarized (1 = Man, 2 = Woman; ‘prefer not to answer’ excluded from analysis)

^b^Race / ethnicity binarized (1 = White, 2 = Asian, Middle Eastern, Aboriginal or Torres Strait Islander, African, Pacific Islander, Other)

^c^Geographic location binarized (1 = Metropolitan, 2 = Rural, Regional, Remote)

^d^Type of Institution binarized (1 = Government/public, 2 = Systemic, Independent/non-government, Other)

**Supplementary Table 2**

*Knowledge Test Items and Answers, and Percentage Answer Endorsed by Educators*

|  | **Question** | **Answers** | **%** |
| --- | --- | --- | --- |
| *Topic: Characteristics of conduct problems* | | |  |
| 1 | Select the correct statement below (choose one) | Genetic factors (e.g., family history of mental health concerns) are the main causes for why certain children display severe behaviour problems | 1.5 |
|  |  | ​​Environmental factors (e.g., home environment) are the main causes for why certain children display severe behaviour problems | 7.7 |
|  |  | **Multiple genetic and environmental factors interact with one another to make it more likely that children display severe behavioural problems**^[[1]](#endnote-1),^^[[2]](#endnote-2)^ | **87.7** |
|  |  | None of the above statements are correct | 3.1 |
| 2 | Which of the following symptoms are all associated with Conduct Problems? | Lack of remorse/guilt, Lack of empathy, poor social skills, hyperactivity, verbal and physical aggression | 16.7 |
|  |  | Defiance towards authority figures, hostility, non-compliance, hyperactivity/impulsivity, verbal and physical aggression | 46.8 |
|  |  | Lack of remorse/guilt, Lack of empathy, Shallow/superficial emotions, uncaring attitudes about performance | 5.9 |
|  |  | **Defiance towards authority figures, hostility, non-compliance, deceitfulness/lying, verbal and physical aggression**^[[3]](#endnote-3),^^[[4]](#endnote-4)^ | **30.6** |
| 27 | Select the statement that is INCORRECT: | Higher emotional literacy is associated with improved academic functioning | 12.8 |
|  |  | Higher emotional literacy is associated with improved social functioning | 4.6 |
|  |  | **Emotional literacy naturally increases and develops over time at similar rates for all children**^[[5]](#endnote-5)^ | **68.2** |
|  |  | Emotional literacy helps children to understand, communicate and regulate their emotions | 14.4 |
| *Topic: Callous-unemotional (CU) traits / limited prosocial emotions (LPE)* | | |  |
| 3 | Which of the following symptoms are associated with Limited Prosocial Emotions | Lack of remorse/guilt, Lack of empathy, poor social skills, hyperactivity, verbal and physical aggression | 33.2 |
|  |  | Defiance towards authority figures, poor social skills, non-compliance, hyperactivity, verbal and physical aggression | 9.8 |
|  |  | **Lack of remorse/guilt, lack of empathy, shallow/superficial emotions, uncaring attitudes about performance**^3^ | **50.1** |
|  |  | Defiance towards authority figures, hostility, non-compliance, deceitfulness/lying, verbal and physical aggression | 6.9 |
| 34 | TRUE OR FALSE: Students with Conduct Problems and Limited Prosocial Emotions require tailored behavioural management strategies because they have unique deficits and needs. | **True**^[[6]](#endnote-6),^^[[7]](#endnote-7)^ | **96.4** |
|  |  | False | 3.6 |
| 35 | Which of the following options is NOT a relevant target behaviour for students with Limited Prosocial Emotions? | Caring behaviours (e.g., comforting others) | 21.0 |
|  |  | **Improved self-confidence**^[[8]](#endnote-8)^ | **31.0** |
|  |  | Recognising and responding to other’s distress | 21.0 |
|  |  | Reparative behaviours (e.g., apologising) | 26.9 |
| *Topic: Evidence-based principles of behavior management* | | |  |
| 4 | Select the correct word to fill in the blank. _______ means that a behaviour has been followed by a favourable consequence, and hence is more likely to happen again in the future. | Modelling | 11.0 |
|  |  | Shaping | 10.0 |
|  |  | **Reinforcement**^[[9]](#endnote-9)^ | **74.9** |
|  |  | Punishment | 4.1 |
| 5 | Select the correct word to fill in the blank. _______ means that a behaviour has been followed by an unfavourable consequence, and hence it is less likely to happen again in the future. | Modelling | 3.1 |
|  |  | Shaping | 9.0 |
|  |  | Reinforcement | 11.8 |
|  |  | **Punishment**^9^ | **76.2** |
| 6 | Positive Reinforcement refers to: | **Introducing a pleasant consequence to increase a behaviour**^9^ | **93.1** |
|  |  | Removing an unpleasant consequence to increase a behaviour | 3.3 |
|  |  | Introducing an unpleasant consequence to reduce a behaviour | 1.8 |
|  |  | Removing something pleasant to reduce a behaviour | 1.8 |
| 7 | Negative Reinforcement refers to: | Introducing a pleasant consequence to increase a behaviour | 1.3 |
|  |  | **Removing an unpleasant consequence to increase a behaviour**^9^ | **20.0** |
|  |  | Introducing an unpleasant consequence to reduce a behaviour | 58.5 |
|  |  | Removing something pleasant to reduce a behaviour | 20.3 |
| 8 | Positive Punishment refers to: | Introducing a pleasant consequence to increase a behaviour | 21.8 |
|  |  | Removing an unpleasant consequence to increase a behaviour | 25.1 |
|  |  | **Introducing an unpleasant consequence to reduce a behaviour**^9^ | **29.0** |
|  |  | Removing something pleasant to reduce a behaviour | 24.1 |
| 9 | Negative Punishment refers to: | Introducing a pleasant consequence to increase a behaviour | 1.5 |
|  |  | Removing an unpleasant consequence to increase a behaviour | 10.8 |
|  |  | Introducing an unpleasant consequence to reduce a behaviour | 42.3 |
|  |  | **Removing something pleasant to reduce a behaviour**^9^ | **45.4** |
| 10 | Generally speaking, what are some of the functions of student misbehaviour? | A. To get attention, assistance, social interactions, control, or sensory stimulation | 8.5 |
|  |  | B. To avoid demands from others, social interactions, activities, or sensory stimulation | 0.5 |
|  |  | **Both A and B**^[[10]](#endnote-10),^^[[11]](#endnote-11)^ | **90.9** |
| 11 | When trying to determine the function of a student’s problematic behaviour, what factors should you consider? | What happened before the problem behaviour (triggers) | 9.2 |
|  |  | What happened after the problem behaviour (reactions) | 0.0 |
|  |  | **What happened before (triggers) and after the problem behaviour (reactions)**^10,11^ | **90.8** |
| 12 | The type of attention students get when misbehaving is often: | A. Immediate | 21.0 |
|  |  | B. Exciting and highly emotional with lots of verbal and non-verbal signs | 1.6 |
|  |  | **Both A and B**^10,11,^^[[12]](#endnote-12)^ | **77.4** |
| 13 | What are some of the ways adults accidentally reinforce or maintain problematic behaviour? | A. Providing inconsistent consequences for misbehaviour | 5.6 |
|  |  | B. Paying attention to attention-seeking misbehaviour | 1.5 |
|  |  | C. Not paying attention to instances of appropriate or prosocial behaviour | 1.5 |
|  |  | B and C | 12.8 |
|  |  | **All of the above**^10,11^ | **78.5** |
| *Topic: Evidence-based strategies of behavior management* | | |  |
| 14 | What is one of the most important things to consider when trying to establish a positive relationship with a student? | Increase the amount of positive attention you give to that student | 25.4 |
|  |  | Loosen the rules and expectations you have for that student | 0.3 |
|  |  | Reduce the amount of negative attention you give to that student | 3.6 |
|  |  | **Generally provide more positive attention than negative attention**^[[13]](#endnote-13)^ | **29.3** |
|  |  | Ensure there is a balance in the amount of positive to negative attention you give to that student | 41.4 |
| 15 | Which of the following statements is NOT true about child-led play? | Provides the child with a sense of autonomy and independence | 6.4 |
|  |  | Fosters a stronger adult-child relationship | 21.5 |
|  |  | **Teaches the child how to be a good leader**^[[14]](#endnote-14),^^[[15]](#endnote-15),^^[[16]](#endnote-16)^ | **46.2** |
|  |  | Provides the child with an opportunity to receive high-quality, positive attention | 25.9 |
| 16 | TRUE OR FALSE: Providing children with labelled and unlabelled praises are equally effective ways of increasing the likelihood of specific behaviours occurring again in the future. | True | 49.7 |
|  |  | **False**^[[17]](#endnote-17),^^[[18]](#endnote-18)^ | **50.3** |
| 17 | Which of the following factors are NOT characteristic of effective praise? | Delivered consistently and immediately after a desirable behaviour | 2.6 |
|  |  | Delivered enthusiastically | 5.1 |
|  |  | Specific in nature | 4.4 |
|  |  | **Used sparingly at first when teaching a new skill**^17,18^ | **87.9** |
| 18 | What is the purpose of describing a child’s appropriate behaviour? | Shows that you are interested in their feelings and allows you to understand the child better | 17.3 |
|  |  | **Helps to keep the child’s attention on the task and shows that you approve of their behaviour**^[[19]](#endnote-19),^^[[20]](#endnote-20)^ | **51.5** |
|  |  | Models good language skills and provides an opportunity to place all the focus on the child and how they are feeling | 31.3 |
| 19 | Which of the following is an effective way to describe an emotion (also known as emotion coaching)? | **You look sad from the way you are frowning and looking down**^[[21]](#endnote-21),^^[[22]](#endnote-22)^ | **85.4** |
|  |  | It is annoying you that the blocks keep falling down... | 11.0 |
|  |  | You’re jealous that your friend got to have a turn before you | 2.1 |
|  |  | You look like you are bored | 1.5 |
| 20 | Which of the following is the best example of a clear direction: | “Can you please pass me the crayons” | 4.6 |
|  |  | “Will you hand me the red block” | 13.1 |
|  |  | **“Please pass me the paintbrushes from the bucket”**^[[23]](#endnote-23),^^[[24]](#endnote-24)^ | **62.6** |
|  |  | “I’d like you to sit still please” | 19.7 |
| 21 | Which of the following is NOT a clear direction: | “Sit in your seat please” | 2.3 |
|  |  | **“Could you take out the art supplies please”**^23,24^ | **81.2** |
|  |  | “Please pick up your rubbish” | 3.6 |
|  |  | “Put your books in a neat pile” | 12.9 |
| 22 | TRUE OR FALSE: Using questions during child-led play is a good way to show that you are interested. | TRUE | 95.1 |
|  |  | **FALSE**^19,20,^^[[25]](#endnote-25)^ | **4.9** |
| 23 | What is negative talk during child-led play? | A. Statements that express disapproval of the child or what s/he is doing | 1.5 |
|  |  | B. Correcting mistakes and providing constructive feedback | 1.0 |
|  |  | C. Sarcastic and/or rude statements towards the child | 5.1 |
|  |  | **All of the above**^23,^^[[26]](#endnote-26)^ | **28.7** |
|  |  | Options A and C | 63.6 |
| 24 | Why should negative talk be avoided? | A. Lowers child’s self-esteem | 4.6 |
|  |  | B. Increases the behaviour you want the child to stop | 0.8 |
|  |  | C. Models rude or inappropriate behaviour to the child | 0.3 |
|  |  | Both A and C | 29.5 |
|  |  | **All of the above**^23,^^[[27]](#endnote-27),^^[[28]](#endnote-28)^ | **64.9** |
| 25 | What types of rewards are most effective for motivating appropriate student behaviours? | **A. Social and tangible rewards depending on the child’s preferences**^[[29]](#endnote-29),^^[[30]](#endnote-30)^ | **43.7** |
|  |  | B. Social rewards (e.g., high-5, praise) | 5.4 |
|  |  | C. Tangible rewards (e.g., stickers, tokens) | 0.8 |
|  |  | Both B and C | 41.9 |
|  |  | None of the above, using rules and limits is the most effective way of motivating appropriate behaviours in students | 8.2 |
| 26 | Which of the following factors are NOT important when using rewards to motivate behaviour? | **Selection of rewards should be changed occasionally as a surprise to the child**^29,^^[[31]](#endnote-31),^^[[32]](#endnote-32)^ | **71.4** |
|  |  | Rewards should be provided immediately and consistently after appropriate behaviour occurs | 8.8 |
|  |  | Selection of rewards should be changed occasionally in consultation with the child | 13.7 |
|  |  | Having a clear idea of which behaviours warrant a reward versus those that do not | 6.2 |
| 28 | Which of the following options is NOT a characteristic of a clear direction? | Direct and specific | 2.1 |
|  |  | **Simultaneously explains why compliance is necessary**^23,24^ | **6.2** |
|  |  | Developmentally appropriate | 64.1 |
|  |  | Polite and respectful | 27.7 |
| 29 | From the options below, select the direction that is BOTH positively stated AND direct: | “You need to stop being so silly” | 2.1 |
|  |  | “No yelling in the classroom” | 6.2 |
|  |  | “Can you use your inside voice” | 64.1 |
|  |  | **“Turn the computer off”** ^23,24^ | **27.7** |
| 30 | What is the most important thing to keep in mind BEFORE giving a clear direction? | **Consider if the child is actively listening and you have time to follow-through if the child does not comply**^23,24^ | **14.1** |
|  |  | Consider if the direction has been followed in the past and if other students have struggled to comply too | 3.3 |
|  |  | Consider if the direction is necessary, if the child is actively listening, and is in a good emotional and physical state to be able to comply | 77.9 |
|  |  | None of the above | 4.6 |
| 31 | Which of the following is most effective when responding to student behaviour that functions to get the child out of following a direction: | **Validate the child’s feelings briefly, then re-direct to the original request. If needed, assist the child in following through with the request**^12,^^[[33]](#endnote-33),^^[[34]](#endnote-34)^ | **90.3** |
|  |  | Offer to compromise and make the direction a bit easier to follow | 5.6 |
|  |  | Send the child to time out for refusing to comply | 2.3 |
|  |  | Ignore the child’s non-compliance and wait for him/her to eventually follow the original direction | 1.8 |
| 32 | Bradley calls out the teacher’s name loudly in class while the teacher is assisting another student. It appears the function of Bradley’s behaviour is to get the teacher’s attention. What is the most effective way of responding to this type of attention-seeking behaviour? | A. Send Bradley to time out for shouting in class | 0.8 |
|  |  | B. Provide Bradley with multiple warnings and then begin placing an ‘X’ next to his name on the board. Each time he calls out, place an X next to his name and remind him that he will get detention if he does not stop shouting | 1.0 |
|  |  | C. Validate Bradley’s desire to get attention then remind him to raise his hand | 20.0 |
|  |  | D. Ignore Bradley’s behaviour and wait for him to stop shouting. Once quiet, praise him for engaging in a positive opposite behaviour (e.g., raising his hand, using quiet, calm voice, waiting patiently) | 4.4 |
|  |  | E. Ignore Bradley’s behaviour and praise positive behaviours in surrounding children (e.g., “I like how Lucy is raising her hand to speak and waiting patiently”) | 6.2 |
|  |  | Options B and D | 4.6 |
|  |  | **Options D and E**^12,33,34^ | **63.1** |
| 33 | For a student that has just successfully  completed timeout and re-joined the class, why is it important to quickly find something positive in their behaviour to praise? | A. Helps to restore the student-teacher relationship | 5.1 |
|  |  | B. Highlights to the student that positive behaviour is rewarded with attention while misbehaviour results in removal of attention (e.g., timeout) | 4.1 |
|  |  | C. Builds the child’s self-esteem back up | 2.1 |
|  |  | **Options A and B**^12,23,33,34^ | **48.8** |
|  |  | Option B and C | 39.8 |

*Note.* Correct answer is indicated in boldface.

**References**

1. Hawes, D. J., Gardner, F., Dadds, M. R., Frick, P. J., Kimonis, E. R., Burke, J. D., & Fairchild, G. (2023). Oppositional defiant disorder. *Nature Reviews Disease Primers*, *9*, 31. <https://doi.org/10.1038/s41572-023-00441-6> [↑](#endnote-ref-1)
2. Fairchild, G., Hawes, D. J., Frick, P. J., Copeland, W. E., Odgers, C. L., Franke, B., ... & De Brito, S. A. (2019). Conduct disorder. *Nature Reviews Disease Primers*, *5*, 43. <https://doi.org/10.1038/s41572-019-0095-y> [↑](#endnote-ref-2)
3. American Psychiatric Association. (2013). *Diagnostic and statistical manual of mental disorders* (5th ed.). <http://doi.org/10.1176/appi.books.9780890425596> [↑](#endnote-ref-3)
4. Kimonis, E. R. & Fleming, G. E. (2018). Disruptive and conduct disorders, delinquency. In T. H. Ollendick, S. W. White, & B. A. White (Eds.), *The Oxford Handbook of Clinical Child and Adolescent Psychology*. Oxford University Press. <https://doi.org/10.1093/oxfordhb/9780190634841.013.27> [↑](#endnote-ref-4)
5. Southam-Gerow, M. A., & Kendall, P. C. (2002). Emotion regulation and understanding: Implications for child psychopathology and therapy. *Clinical Psychology Review*, *22*(2), 189-222. <https://doi.org/10.1016/S0272-7358(01)00087-3> [↑](#endnote-ref-5)
6. Frick, P. J. (2012). Developmental pathways to conduct disorder: Implications for future directions in research, assessment, and treatment. *Journal of clinical child & adolescent psychology*, *41*(3), 378-389. <https://doi.org/10.1080/15374416.2012.664815> [↑](#endnote-ref-6)
7. Levine, R. S., Smith, K., & Wagner, N. J. (2022). The impact of callous-unemotional traits on achievement, behaviors, and relationships in school: A systematic review. *Child Psychiatry & Human Development*. <https://doi.org/10.1007/s10578-022-01344-5> [↑](#endnote-ref-7)
8. Frick, P. J., Ray, J. V., Thornton, J. C., & Kahn, R. E. (2014). Can callous-unemotional traits enhance the understanding, diagnosis, and treatment of serious conduct problems in children and adolescents? A comprehensive review. *Psychological Bulletin, 140*, 1-57. <https://doi.org/10.1037/a0033076> [↑](#endnote-ref-8)
9. Skinner, B. F. (1953). *Science and human behavior*. Free Press. [↑](#endnote-ref-9)
10. Beavers, G. A., Iwata, B. A., & Lerman, D. C. (2013). Thirty years of research on the functional analysis of problem behavior. *Journal of Applied Behavior Analysis*, *46*(1), 1-21. <https://doi.org/10.1002/jaba.30> [↑](#endnote-ref-10)
11. Kestner, K. M., & St. Peter, C. C. (2018). Functional analysis and challenging behavior. In C. McNeil, L. Quetsch, & C. Anderson (Eds.), *Handbook of Parent-Child Interaction Therapy for Children on the Autism Spectrum* (pp. 169-187). <https://doi.org/10.1007/978-3-030-03213-5_10> [↑](#endnote-ref-11)
12. Fernandez, M. A., Gold, D. C., Hirsch, E., & Miller, S. P. (2015). From the clinics to the classrooms: A review of teacher-child interaction training in primary, secondary, and tertiary prevention settings. *Cognitive and Behavioral Practice*, *22*(2), 217-229. <https://doi.org/10.1016/j.cbpra.2014.01.004> [↑](#endnote-ref-12)
13. Kincade, L., Cook, C., & Goerdt, A. (2020). Meta-analysis and common practice elements of universal approaches to improving student-teacher relationships. *Review of Educational Research*, *90*(5), 710-748.

    <https://doi.org/10.3102/0034654320946836> [↑](#endnote-ref-13)
14. McNeil, C. B., & Hembree-Kigin, T. L. (2010). Overview of Parent-Child Interaction Therapy. In C. B. McNeil & T. L. Hembree-Kigin (Eds.), *Parent-Child Interaction Therapy* (pp. 3-16). Springer. [↑](#endnote-ref-14)
15. Tiano, J. (2010). Teacher–Child Interaction Therapy for Preschool Classrooms. In C. B. McNeil & T. L. Hembree-Kigin (Eds.), *Parent-Child Interaction Therapy* (pp. 385-391). Springer. [↑](#endnote-ref-15)
16. Webster-Stratton, C., & Reid, M. J. (2010). Parents, teachers, and therapists using child-directed play therapy and coaching skills to promote children's social and emotional competence and build positive relationships. In C. E. Schaefer (Ed.), *Play therapy for preschool children* (pp. 245–273). American Psychological Association. [https://doi.org/10.1037/12060-012](https://psycnet.apa.org/doi/10.1037/12060-012) [↑](#endnote-ref-16)
17. Chalk, K., & Bizo, L. A. (2004). Specific praise improves on‐task behaviour and numeracy enjoyment: A study of year four pupils engaged in the numeracy hour. *Educational psychology in Practice*, *20*(4), 335-351. <https://doi.org/10.1080/0266736042000314277> (but see also: Leijten, P., Thomaes, S., de Castro, B. O., Dishion, T. J., & Matthys, W. (2016). What good is labeling what's good? A field experimental investigation of parental labeled praise and child compliance. Behaviour Research and Therapy, 87, 134-141. <https://doi.org/10.1016/j.brat.2016.09.008>) [↑](#endnote-ref-17)
18. Ennis, R. P., Royer, D. J., Lane, K. L., & Dunlap, K. D. (2020). Behavior-specific praise in pre-K–12 settings: Mapping the 50-year knowledge base. *Behavioral Disorders*, *45*(3), 131-147. <https://doi.org/10.1177/0198742919843075> [↑](#endnote-ref-18)
19. McNeil, C. B., & Hembree-Kigin, T. L. (2010). Teaching Child-Directed Interaction. In C. B. McNeil, & T. L. Hembree-Kigin (Eds.), *Parent-Child Interaction Therapy* (pp. 49-76). Springer. [↑](#endnote-ref-19)
20. Tempel, A. B., Wagner, S. M., & McNeil, C. B. (2013). Behavioral parent training skills and child behavior: The utility of behavioral descriptions and reflections. *Child & Family Behavior Therapy*, *35*(1), 25-40. <https://doi.org/10.1080/07317107.2013.761009> [↑](#endnote-ref-20)
21. Gottman, J. M., & DeClaire, J. (1996). *The heart of parenting: Raising an emotionally intelligent child*. Simon & Schuster. [↑](#endnote-ref-21)
22. Katz, L. F., Maliken, A. C., & Stettler, N. M. (2012). Parental meta‐emotion philosophy: A review of research and theoretical framework. *Child Development Perspectives*, *6*(4), 417-422. <https://doi.org/10.1111/j.1750-8606.2012.00244.x> [↑](#endnote-ref-22)
23. McNeil, C. B., & Hembree-Kigin, T. L. (2010). Teaching Parent-Directed Interaction. In C. B. McNeil, & T. L. Hembree-Kigin (Eds.), *Parent-Child Interaction Therapy* (pp. 103-132). Springer. [↑](#endnote-ref-23)
24. Fernandez, M. A., Adelstein, J. S., Miller, S. P., Areizaga, M. J., Gold, D. C., Sanchez, A. L., ... & Gudiño, O. G. (2015). Teacher-child interaction training: A pilot study with random assignment. *Behavior Therapy*, *46*(4), 463-477. <https://doi.org/10.1016/j.beth.2015.02.002> [↑](#endnote-ref-24)
25. Tempel, A. B., Wagner, S. M., & McNeil, C. B. (2009). Parent-child interaction therapy and language facilitation: The role of parent-training on language development.*The Journal of Speech and Language Pathology – Applied Behavior Analysis, 3*(2-3), 216–232. <https://doi.org/10.1037/h0100241> [↑](#endnote-ref-25)
26. Filcheck, H. A., McNeil, C. B., Greco, L. A., & Bernard, R. S. (2004). Using a whole‐class token economy and coaching of teacher skills in a preschool classroom to manage disruptive behavior. *Psychology in the Schools*, *41*(3), 351-361. <https://doi.org/10.1002/pits.10168> [↑](#endnote-ref-26)
27. Caspi, A., Moffitt, T. E., Morgan, J., Rutter, M., Taylor, A., Arseneault, L., Tully, L., Jacobs, C., Kim-Cohen, J., & Polo-Tomas, M. (2004). Maternal expressed emotion predicts children's antisocial behavior problems: Using monozygotic-twin differences to identify environmental effects on behavioral development. *Developmental Psychology, 40*(2), 149–161. [https://doi.org/10.1037/0012-1649.40.2.149](https://psycnet.apa.org/doi/10.1037/0012-1649.40.2.149) [↑](#endnote-ref-27)
28. Pasalich, D. S., Dadds, M. R., Hawes, D. J., & Brennan, J. (2011). Assessing relational schemas in parents of children with externalizing behavior disorders: Reliability and validity of the Family Affective Attitude Rating Scale. *Psychiatry Research*, *185*(3), 438-443. <https://doi.org/10.1016/j.psychres.2010.07.034> [↑](#endnote-ref-28)
29. Kazdin, A. E. (2008). *Parent management training: Treatment for oppositional, aggressive, and antisocial behavior in children and adolescents*. Oxford University Press. [↑](#endnote-ref-29)
30. Kohls, G., Peltzer, J., Herpertz‐Dahlmann, B., & Konrad, K. (2009). Differential effects of social and non‐social reward on response inhibition in children and adolescents. *Developmental science*, *12*(4), 614-625. <https://doi.org/10.1111/j.1467-7687.2009.00816.x> [↑](#endnote-ref-30)
31. Ivy, J. W., Meindl, J. N., Overley, E., & Robson, K. M. (2017). Token economy: A systematic review of procedural descriptions. *Behavior Modification*, *41*(5), 708-737. <https://doi.org/10.1177/0145445517699559> [↑](#endnote-ref-31)
32. Ackerman, K. B., Samudre, M., & Allday, R. A. (2020). Practical components for getting the most from a token economy. *Teaching Exceptional Children*, *52*(4), 242-249. <https://doi.org/10.1177/0040059919892022> [↑](#endnote-ref-32)
33. Lyon, A. R., Gershenson, R. A., Farahmand, F. K., Thaxter, P. J., Behling, S., & Budd, K. S. (2009). Effectiveness of teacher-child interaction training (TCIT) in a preschool setting. *Behavior Modification*, *33*(6), 855-884.

    <https://doi.org/10.1177/0145445509344215> [↑](#endnote-ref-33)
34. Tiano, J. D., & McNeil, C. B. (2006). Training Head Start teachers in behavior management using Parent-Child Interaction Therapy: A preliminary investigation.*Journal of Early and Intensive Behavior Intervention, 3*(2), 220–233. <https://doi.org/10.1037/h0100334> [↑](#endnote-ref-34)
